# Form and content of Jamaican cannabis edibles

**DOI:** 10.1186/s42238-021-00079-9

**Published:** 2021-07-10

**Authors:** Carole M. Lindsay, Wendel D. Abel, Erica E. Jones-Edwards, Paul D. Brown, Khalia K. Bernard, Tainia T. Taylor

**Affiliations:** 1 Faculty of Medical Sciences Teaching and Research Complex (FMSTRC), University of the West Indies, Mona, Kingston, Jamaica; 2 Department of Community Health and Psychiatry, University of the West Indies, Mona, Kingston, Jamaica

**Keywords:** Cannabis use, Edibles, Labeling, Safety, Potency, Jamaica

## Abstract

**Background:**

In 2017, the Jamaican government banned the sale of cannabis-infused edibles after reports of over-intoxication in adults and children. There is a general lack of public awareness regarding the risk involved with edible dosage. Vandrey et al. in 2015 reported that random cannabis edibles sampled from dispensaries in California and Washington in the USA failed to meet the basic labeling standards for pharmaceuticals (Vandrey et al.; JAMA 2015). This study aims to measure the levels of THC and CBD in a variety of edibles available locally in order to establish current cannabinoid content and to report on safety and packaging. This study is deemed necessary as no such study has been done to measure the potency levels of edibles and to raise awareness of the potential risk to children.

**Methods:**

Forty-five cannabis-infused edible items were collected as convenience samples over a 4-year period (2014–2018) and analyzed. The QuEChERS technique (modified) was used to extract cannabinoids from each item. The extracts were then derivatized with MSTFA prior to analysis by gas chromatography-mass spectrometry (GC-MS). The descriptive statistics were calculated using the Statistical Package for Social Sciences—SPSS Software. Descriptive statistics presented include the mean, median, standard deviation, and range for each product category. The distribution of data with a box and whisker plot and frequency of THC to CBD ratios with a histogram was also presented.

**Results:**

Edibles on the Jamaican market comprise baked goods, candies, frozen foods, and beverages. Eighty-six percent of samples were poorly labeled and failed to meet basic labeling requirements. None of the packages were observed to be child-proof. THC levels ranged from 0.01 to 99.9 mg per product while CBD levels ranged from 0.001 to 69.2 mg per product. The highest THC and CBD levels were detected in cookies and brownies. Thirty percent of the samples had THC levels greater than the recommended 10 mg THC per serving.

**Conclusion:**

The lack of proper labeling and the wide range of THC levels in cannabis edibles raises public health concerns for all consumers including inexperienced persons who may be at a greater risk of overdosing. Concern must also be raised over the possibility that the attractive and tasty ways in which the drug is being presented might tempt young children and teens to take cannabis.

Impact statement

It is hoped that this information will raise public awareness of the current potential danger these edibles pose to children and inexperienced users and that policymakers will see the need for the imposition of suitable regulations.

## Introduction

Cannabis is the most widely used illicit substance globally with 5.6% of adults and youth reporting use (United Nations Office on Drugs and Crime, 2018). For centuries, the drug has been used across cultures for medicinal, recreational, and sacramental purposes (Abel et al. 2011). In recent years, there have been accelerated changes in drug policy in regard to cannabis globally (Abel et al. 2011; Oshi et al. 2019). Such changes include decriminalization which involves the removal of criminal penalties for possession of a small amount of the substance or legalization of cannabis (Abel et al. 2011).

In 2015, Jamaica amended its Dangerous Drug Act resulting in the removal of the criminal penalty for possession of less than 2 ounces of cannabis. Additionally, citizens are allowed to grow no more than 5 plants in their homes, and Rastafarians are allowed to use cannabis freely for religious purposes (Government of Jamaica: Ministry of Justice 2015).

In 2015, Jamaica established a Cannabis Licensing Authority with the responsibility to develop a licensing regimen and regulatory systems. Before the body was fully established, there was an explosion of edibles on the Jamaican market and many of these products were unregulated with no clear standards to ensure consistent dosing, quality, and the safety of the products. It is in this context that increased efforts were made to monitor the range and constituents of edibles on the Jamaican market. In May 2017, the Jamaican Ministry of Justice banned the sale and sampling of these products from all festivals and entertainment events in Jamaica following the National Council on Drug Abuse (NCDA) 2016 report on several cases of over-intoxication from edibles (Virtue 2017).

A National Drug Prevalence Survey conducted in Jamaica in 2016 by NCDA revealed that the prevalence of persons who use cannabis at least once in a lifetime was 28.3% and the prevalence of current use was 15.8% (Younger-Coleman et al. 2017). School surveys also report that 20.7% of students 12–18 years old have used cannabis at least once in their lifetime while 11.7% have used it within the past month (NCDA 2013). Furthermore, studies also suggest that the mean age of initiation is around 15–16 (Oshi et al. 2018; Bernard et al. 2017).

The cannabinoid tetrahydrocannabinol (THC) is the main psychoactive component in cannabis. Another cannabinoid of growing significance is the non-psychoactive cannabidiol (CBD). The cannabis plant is recognized for its therapeutic and recreational properties with an evolving body of literature indicating the possible medicinal properties of both THC and CBD.

Cannabinoids are produced in cannabis as their carboxylic acid derivatives and include ∆-9-tetrahydrocannabinolic acid (THCA) and cannabidiolic acid (CBDA), which are converted to their neutral counterparts (THC and CBD respectively) when heated, exposed to sunlight, or in long-term storage (MacCallum and Russo 2018; Taura et al. 2007).

Depending on the modes of administration, temporary psychotic symptoms (Wilkinson et al. 2014) and behavioral impairment (Shrivastava et al. 2011; Grotenhermen 2007) are linked to varying levels of THC. Regarding cannabis toxicity, there is enough experimental evidence to show that cannabis is not particularly lethal, and no cannabis-related deaths due to acute physical toxicity have been reported (Barrus et al. 2016; Grotenhermen 2007).

Cannabis may be administered by several routes including inhalation, topical, and oral use (Borodovsky et al. 2017). Smoking of dried cannabis plants has traditionally been the preferred route of administration. Cannabis edibles are food products infused with cannabis that are administered orally and include a wide range of products such as candies, baked products, lozenges, and beverages. THC is fat-soluble and, when heated, is easily extracted, along with other cannabinoids, in oils and butter (Barrus et al. 2016). Edibles like hard candies, which do not require baking or cooking during preparation, are usually made from cannabis tinctures. Tinctures are liquid cannabis extracts made from solvents such as alcohol or glycerol (Barrus et al. 2016).

A major distinguishing factor between cannabis inhalation and oral administration is that cannabis smoking introduces THC to the bloodstream in the lungs and this THC reaches the brain in seconds, while the oral consumption of cannabis-infused edibles introduces cannabinoids to the bloodstream more slowly through the digestive system (Barrus et al. 2016).

Once digested, THC is metabolized in the liver to the more potent 11-hydroxy-THC (Huestis 2007). Peak behavioral and physiologic effects happen within minutes of smoking, while responses post oral consumption have a slower onset and greater variability, as well as longer-lasting effects peaking at 30 min after consumption and lasting for up to 3.5 h (Grotenhermen 2003). This delayed onset often results in the ingestion of a large amount of THC.

The global cannabis edible market has seen significant growth in recent years and is projected to grow substantially over the next 5 years. As the trend becomes more popular, an extensive array of edibles that are either commercially prepared or homemade have become available on the market (Barrus et al. 2016). Several factors have contributed to the expansion of the edibles market: they can be produced at home, they are convenient to transport and use, and there is the perception that edibles are more relaxing than inhaled cannabis. It is generally believed that edibles do not present the same health challenges as does smoking cannabis and there is a longer duration of action associated with the use of edibles (Vandrey et al. 2015).

Despite the growing acceptance of cannabis edibles, extant research has shown that a lack of understanding of safe use is associated with inconsistent and often deleterious health effects (Doran and Papadopoulos 2019; Barrus et al. 2016; Vandrey et al. 2015). Over-intoxication can result in vomiting, physical and cognitive impairment, dizziness, anxiety, paranoia, and delusions (Barrus et al. 2016).

In many jurisdictions, producers of edibles have been able to circumvent regulatory systems and this poses challenges to policymakers worldwide (Barrus et al. 2016). One of the major challenges with edibles is that the onset of action is delayed with ingestion as compared to inhalation of cannabis, and this may lead to users overdosing accidentally in order to feel the effects faster (Barrus et al. 2016). Additionally, targeted marketing strategies have led to an increase in popularity among the youth (Borodovsky et al. 2017). The rising popularity of edibles has resulted in an increase in incidences of unintentional cannabis exposures in children 9 years of age and younger (Wang et al. 2014).

In November 2018, the NCDA highlighted significant concerns raised by physicians in Jamaica, reporting an increase in the number of infants who have turned up for emergency care due to accidental ingestion of cannabis edibles (Taylor 2018). Anecdotal reports suggest that in all reported cases of overconsumption of edibles in Jamaica, the THC levels in the edibles were not determined. This raised concern about the levels and variability of THC in locally available cannabis-infused foods and the potential risks. Currently, there are no laws in place in Jamaica governing the production, labeling, and safe use of edibles thereby creating a space for over-intoxication.

As such, this study aims to identify and measure the levels of THC and CBD in a variety of edibles available locally in order to establish cannabinoid content. It also seeks to examine packaging for adequate labeling and safety. Such a study would be the first of its kind to be conducted in Jamaica.

## Methods and materials

### Materials

Certified reference material for THC, CBD, THCA-A, CBDA, CBN, THC-d3, CBD-d3, and CBN-d3 was purchased from Cerilliant Corporation (Round Rock, TX). All standards were analytical grade and were provided as either 1 mg/mL or 100 μg/mL (THC-d3, CBD-d3, CBN-d3) solution in methanol or acetonitrile. All solvents used were HPLC grade and purchased from Sigma-Aldrich (Missouri, USA).

### Sample size and sampling strategy

Forty-five edible cannabis-infused products were used for this study. These items were collected over a 4-year period (2014–2018) through convenience sampling, that is, products were obtained either through a donation from budding entrepreneurs, confiscation from high school students, purchased on the black market at various events, or items for sampling at cannabis seminars. The details of the sample sources are as follows:

**Table:** 

Source	Method of collection	Products collected	Year of collection	Legal status
High schools	Submitted for testing	Baked goods	2014	Illegal
	Submitted for testing	Candies	2015	Decriminalized
	Submitted for testing	Baked goods and candies	2017	Decriminalized
Rastafarian	Submitted for testing	Baked goods and candies	2016	Decriminalized
Cannabis seminars	Donated	Baked Goods	2016	Decriminalized
	Donated	Baked goods, candies, and preserves	2017	Decriminalized
Entertainment events	Bought by patrons	Baked goods	2016	Decriminalized
	Bought by patrons	Baked goods and beverages	2018	Decriminalized
University students	Donated	Baked goods and candies	2016	Decriminalized
	Donated	Baked goods and preserves	2018	Decriminalized
Cannabis companies	Donated	Baked goods and candies	2016	Decriminalized
	Donated	Candies	2017	Decriminalized
University student vendor	Donated	Bread	2017	Decriminalized
Oregon dispensary	Purchased	Candies	2017	Decriminalized
	Purchased	Chocolates	2017	Decriminalized

The sample size obtained and the non-probability sampling technique are a direct consequence of the fact that marijuana is illegal in Jamaica. In 2015, marijuana was decriminalized in Jamaica, and the majority of the items were obtained after this period.

Upon receipt of the products, they were assessed and placed into one of six categories namely baked goods, beverages, candies, chocolates, frozen foods, and preserves. The operational definitions guiding this classification process were as follows:

Baked Goods—cannabis-infused products made with cannabis butter or cannabis oil. They included brownies, bread, oatmeal cookies, chocolate chip cookies, carrot cake, fruit cake, coconut chocolate chip cookie, cupcakes, and Danish pastry.

Beverages—cannabis-infused drinks and included coffee and grape-flavored wine.

Cannabis candies—cannabis-infused candies and included gummy bears, chocolate candy, busta, kush candy, jewel candy, mango lollipop, tamarind ball, lime lollipop, and peanut cake, while chocolate bars were grouped separately.

Cannabis-infused ice cream and stewed June plum were categorized as frozen foods and preserves respectively.

### Sample pre-treatment

Whole candies, cookies, brownies, and slices of cakes and bread were ground to a fine powder using a Proctor-Silex Coffee Grinder, while gummy candies and chocolates were cut into small pieces (approx. 0.5–2 mm). All samples were stored at − 20 °C until required for extraction.

### Sample extraction

A modification of the QuEChERS (Quick, Easy, Cheap, Effective, Rugged, and Safe) technique originally published by Wang et al. (2016) was used to extract cannabinoids from cannabis-containing foods (UCT, LLC 2015). The extracts were then diluted in preparation for instrumental analysis.

One gram of pretreated sample was weighed in a 50 mL silanized centrifuge tube. Ten milliliters of distilled water was then added and the mixture vortexed for 30 s. Ten milliliters of acetonitrile with 1% acetic acid was then added, and the mixture vortexed at medium speed for 1 min and shaken for an hour on a horizontal shaker at 150 rpm. Four grams of anhydrous MgSO_4_ + 1g NaCl (extraction salt) was added and the mixture vortexed for 1 min, centrifuged at 3000 rpm for 5 min, and the supernatant transferred to a silanized culture tube. The supernatant was then evaporated to dryness at 40 °C, under a gentle stream of nitrogen and the dried extract reconstituted in 1 mL of hexane to ethyl acetate (1:1). Serial dilutions of extracts were performed ranging from 50 to 400 times.

A volume of 100 μL of the diluted sample was removed to a silanized autosampler vial along with 50 μL of the working solution of internal standard (0.2 ppm) before drying at 40 °C under nitrogen. The dried extracts were derivatized by adding 100 μL MSTFA and heating at 70 °C for 30 min. One microliter of the derivatized extract was injected into the GC–MS system.

### Preparation of standard solutions

A standard stock solution containing THC, CBD, CBN, CBDA, and THCA-A each at a concentration of 10 μg/mL was prepared in methanol. Working solutions at concentrations of 10, 100, and 1000 ng/mL were subsequently prepared by diluting the standard stock solution with methanol and stored at − 20 ^°^C until needed for analysis. The internal standard working solutions (THC-d3, CBD-d3, CBN-d3) were prepared at a concentration of 200 ng/mL in methanol.

Calibrators containing THC, CBD, THCA-A, CBDA, and CBN at concentrations equivalent to 0.1, 0.25, 0.5, 1.0, 2.5, 5, 10, and 20 μg/g were prepared in duplicate in cannabis free brownie. The quality control sample consisted of a brownie containing 33.0 mg of THC. Calibrators and quality control samples were treated and processed in the same manner as test samples.

### Instrumentation

GC-MS analysis was performed using an Agilent 7890 Gas Chromatograph equipped with a 7683 Series Autosampler and a 5975 Mass Selective Detector (MSD) using the Chemstation software. Analyte separation was achieved on a fused silica capillary column HP-5MS (30 m × 250 μm i.d. × 0.25 μm film thickness). Helium was used as the carrier gas at a flow rate of 1.0 ml/min. The inlet temperature was set at 250 °C, and samples were injected in the splitless mode. The oven temperature was programmed at 80 °C (hold for 2 min) followed by an increase to 290 °C at a rate of 20 °C/min and held for 2 min. The total run time was 14.50 min with a solvent delay of 3 min.

The mass spectrometer was operated with the electron energy set at 70 eV. The retention times and characteristic mass fragments of the silyl derivatives of the cannabinoids were determined by recording the electron impact (EI) spectra in the total ion monitoring mode (scan range m/z 50–550). For quantitative analysis, the chosen characteristic mass fragments were monitored in the selected-ion-monitoring (SIM) mode: m/z 371,315, 386 for THC, m/z 390, 337, 301 for CBD, m/z 491,493, 492 for CBDA, m/z 487, 489, 488 for THCA, m/z 367, 368, 382 for CBN, m/z 374 for THC-d3, m/z 393 for CBD-d3, and m/z 370 for CBN-d3 (quantitative ions are in bold).

### Method validation

Prior to application to samples, the method was validated for limits of detection and quantification, recovery, linearity, precision, and accuracy according to SWGTOX criteria (SWGTOX 2013).

#### Limits of detection and quantification (LOD and LOQ)

LOD and LOQ were determined in both candy and brownie matrices by using the standard deviation (S.D.) of the mean noise level over the retention time window of each analyte as follows: LOD = 3 × S. D and LOQ = 10 × S.D.

#### Recovery

Recovery samples were prepared by spiking blank brownie and candy samples with cannabinoid standards at 0.25 μg/g, 5 μg/g, and 20 μg/g. Using three replicates at each of the concentration levels, the absolute recoveries were calculated by comparing the peak areas of each cannabinoid obtained from spiked edible samples (brownie and candy) with those found after the direct injection of standard solutions at the same concentrations.

#### Linearity

Calibration curves were obtained from matrix-matched standard solutions at eight different concentrations for each cannabinoid from 0.1 μg/g to 20 μg/g.

#### Precision and accuracy

To test accuracy (recovery), spiked samples were prepared by adding three levels (low, medium, and high) of known concentrations of standards into three sets (n = 3) of replicates of blank edible samples (brownie and candy) giving a total of 12 samples per edible which were then extracted and quantified. For repeatability, samples were spiked similarly but with 6 sets (*n* = 6) of replicates and expressed as the relative S.D. (% RSD) of the calculated concentrations. Accuracy was expressed as the relative error of the calculated concentrations.

### Statistical analysis

The descriptive statistics were calculated using the “Statistical Product and Service Solutions”—SPSS software. SPSS was also used to analyze the distribution of data with a box and whisker plot and the frequency of THC: CBD ratios with a histogram.

## Results

### Method validation

**Fig. 1:**
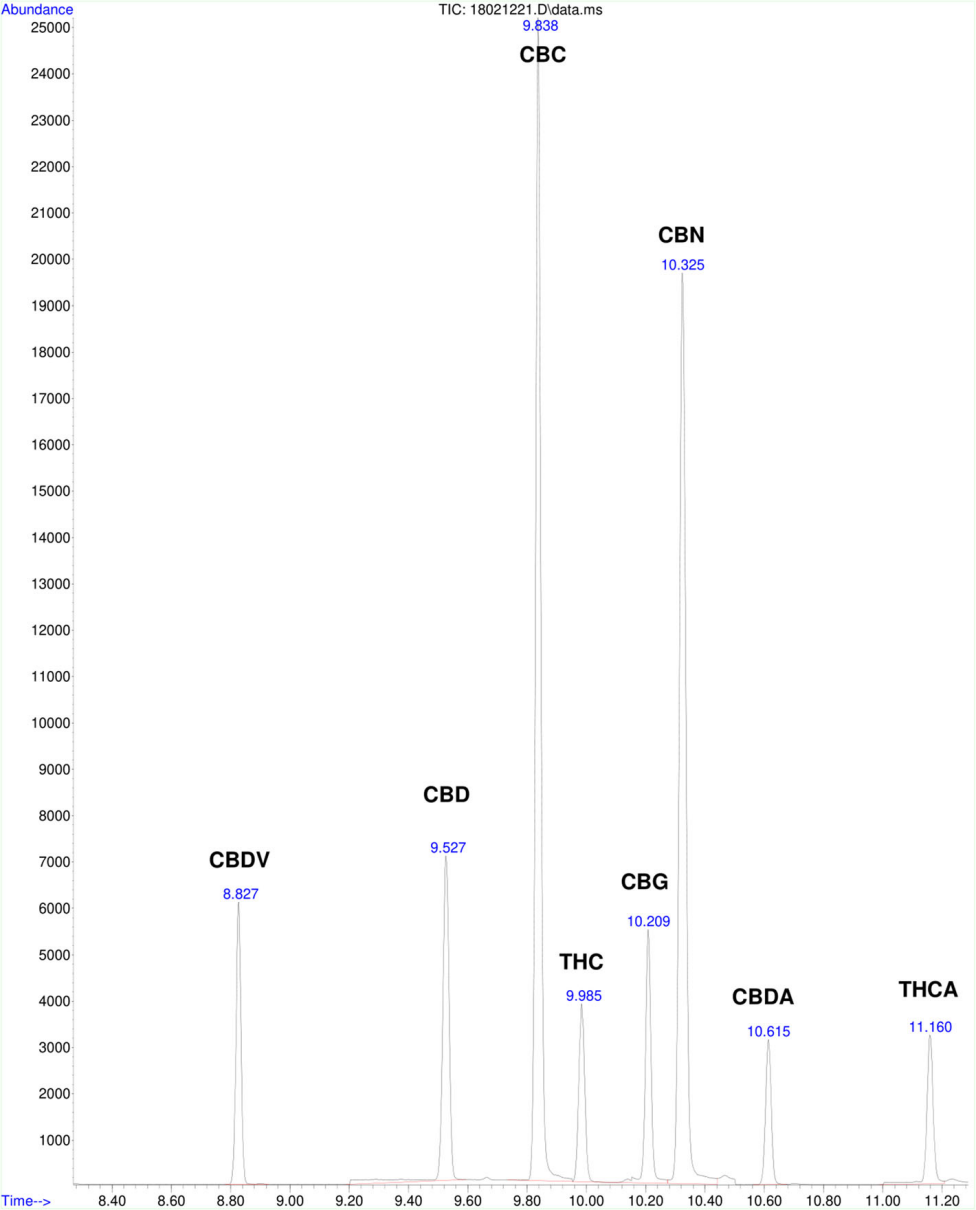
Chromatogram of cannabinoids in a calibrator prepared in a blank brownie. The retention times for cannabinoids shown in the chromatogram are as follows: cannabidivarin (CBDV)-8.827 min, cannabidiol (CBD)—9.527 min, cannabichromene (CBC)—9.838 min, ∆-9-tetrahydrocannabinol (THC)—9.985 min, cannabigerol (CBG)—10.209 min, cannabinol (CBN)—10.325 min, cannabidiolic acid (CBDA)—10.615 min, and ∆-9-tetrahydrocannabinolic acid (THCA)-11.160 min

**Fig. 2:**
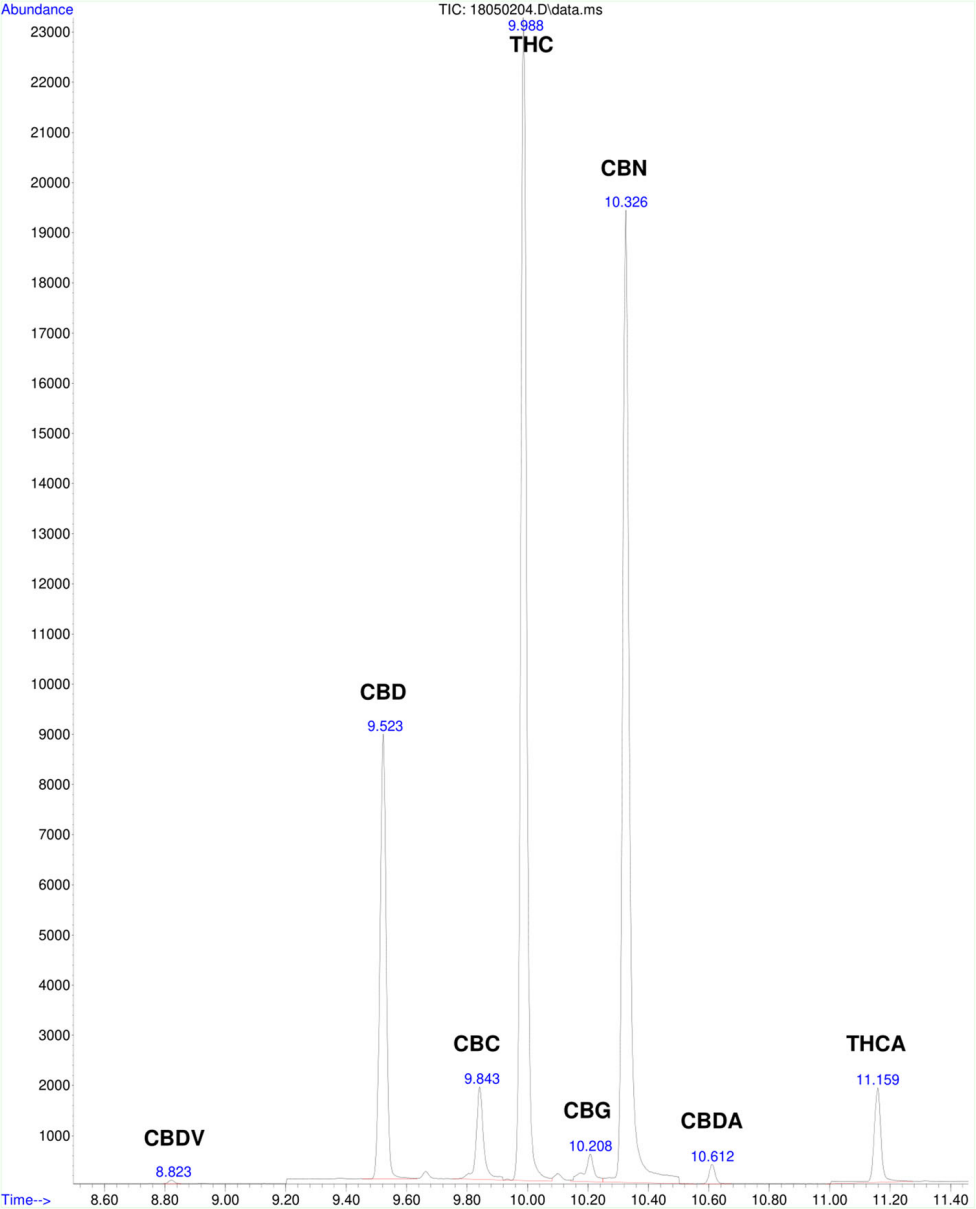
Chromatogram of cannabinoids in a cookie sample. The retention times for the cannabinoids shown in the chromatogram are as follows: CBDV-8.823 min, CBD-9.523 min, CBC-9.843 min, ∆-9-THC-9.988 min, CBG-10.208 min, CBN-10.326 min, CBDA- 10.612 min, and ∆-9-THCA-11.159 min

Cannabinoids were quantified using a reliable and efficient method obtained and modified from United Nations Office on Drugs and Crime (2013). Chromatograms for a calibrator prepared in blank brownie and cookie sample are shown in Fig. 1 and Fig. 2 respectively.

Figure 1 shows the chromatogram of a typical calibrator sample for the separation of the trimethylsilyl derivatives of CBDV, CBD, CBC, ∆-9-THC, CBG, CBN, CBDA, and ∆-9-THCA established by chromatographing pure standards. Under these run conditions, all 8 cannabinoids were well separated in less than 12 min.

Figure 2 displays the chromatogram of a typical cookie sample run under the same conditions as the calibrators. For this cookie sample, 8 cannabinoids were identified.

The accuracy, precision, recovery, LOD, LOQ, and correlation coefficient can be found in Tables 1 and 2 for brownies and candies respectively. Table 1 shows that the method was able to detect and quantify the cannabinoids in brownies at levels lower than 0.03 μg/g. The precision and accuracy for each analyte were below the 20% acceptable limit recommended by SWGTOX 2013 while the recoveries all exceeded 100%. The correlation coefficients were all at least 0.99 and less than 1.0.

**Table 1:** Method validation parameters for gas chromatography mass spectrometry analysis (GCMS) of cannabinoids in brownies

Analyte	Precision (% CV)	Accuracy (%)	Recovery (%)	LOD (μg/g)	LOQ (μg/g)	Correlation coefficient
**THC**	**9.79**	**± 16.54**	**119.81**	**0.001**	**0.005**	**0.9942**
**CBD**	**19.50**	**± 4.39**	**104.59**	**0.008**	**0.025**	**0.9919**
**CBN**	**9.41**	**± 12.92**	**114.83**	**0.005**	**0.015**	**0.9964**

The method validation parameters for GCMS analysis of cannabinoids in brownies (n = 10). Coefficient of variation (CV), limit of detection (LOD), limit of quantification (LOQ)

**Table 2:** Method validation parameters for gas chromatography mass spectrometry analysis (GCMS) of cannabinoids in candy

Analyte	Precision (% CV)	Accuracy (%)	Recovery (%)	LOD (μg/g)	LOQ (μg/g)	Correlation coefficient
**THC**	**5.87**	**± 0.96**	**99.05**	**0.005**	**0.015**	**0.9994**
**CBD**	**7.63**	**± 3.74**	**96.39**	**0.020**	**0.061**	**0.9871**
**CBN**	**0.62**	**± 12.13**	**113.81**	**0.020**	**0.060**	**0.9893**

Table 2 shows the method validation parameters for GCMS analysis of cannabinoids in candy. The number of candies analyzed was 13. Coefficient of variation (CV), limit of detection (LOD), limit of quantification (LOQ)

The method was able to detect and quantify the cannabinoids in candies at levels lower than 0.06 μg/g. The precision and accuracy for each analyte were below the 20% acceptable limit recommended by SWGTOX 2013 while the recoveries all exceeded 100%. The correlation coefficients were all greater than 0.98 but less than 1.0 (Table 2).

### Description of the sample

Among the 45 items used in this study, the majority were baked goods (56%). This was followed by candies (29%) and chocolates (7%). Only one frozen food sample (ice cream) and one preserve (stewed June plum) were obtained.

### Packaging and labeling

We examined the packaging of all samples and found that only 6 of the 41 local products collected were labeled. The six labeled products were collected in the post-decriminalization period (2016–2018), and though the presence of THC was indicated, only four of the six stated the actual amount (milligrams) of THC present. Only two of the labeled products, a chocolate chip cookie and a hard candy, had consumption instructions, outlining the recommended serving size and frequency of eating. For all remaining thirty-nine samples, standard labeling requirements including product name, list of ingredients, name, and address of the manufacturer, lot identification, storage conditions, expiration date, and instructions for use, were missing. The packaging for most of the samples (85%) did not indicate the presence of THC; consequently, making these products visually indistinguishable from their non-cannabis counterparts. We were unable to accurately comment on the packaging of Oregon samples as they were donated by a consumer and were not received in their original packaging.

### THC and CBD content of edibles

**Fig. 3:**
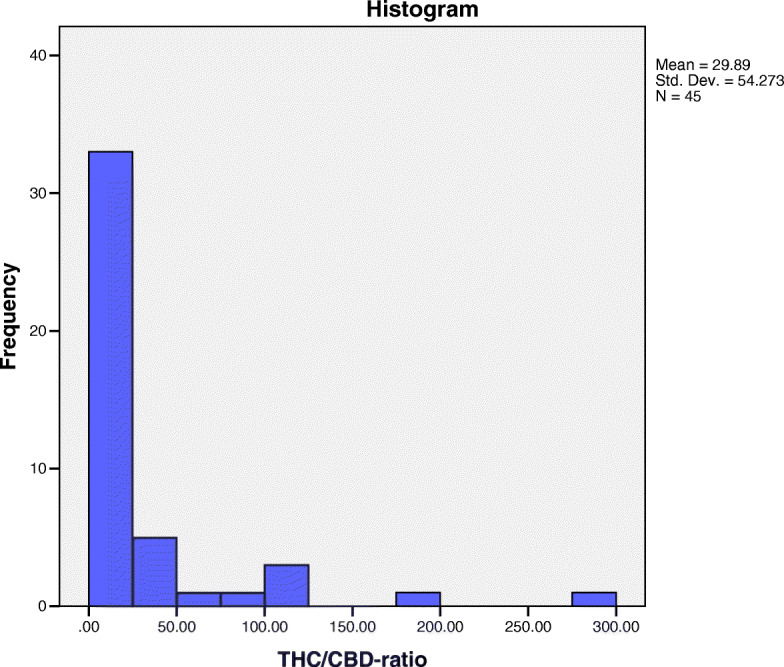
Frequency histogram showing the distribution of THC/CBD ratios in cannabis edibles tested. Each value on the x-axis represents a THC ratio to a CBD value of 1

The ratio of THC to CBD was examined in the test products, and it was found that the majority (87 %) had higher THC to CBD ratios, ranging from 2:1 to 285:1 with the median ratio being 8:1 (Fig. 3). The THC levels of the edible products ranged from a minimum of 0.01 mg/product to a maximum of 99.9 mg/product while CBD levels ranged from a minimum of 0.001 mg/sample to 69.2 mg/sample. A baked product (oatmeal cookie) had the highest THC (99.9 mg) and CBD (69.2 mg) levels; it had relatively equal amounts of THC and CBD, with a THC to CBD ratio of 1:0.7.

Table 3 shows that products having the highest THC levels were baked goods and the lowest THC levels were detected in beverages. Baked products and candies had the highest sample sizes, and therefore, median values were compared. The sample size for all other categories was too small for a meaningful comparison to be made. In baked products, THC levels ranged from 0.8 mg to 99.9 mg with a median of 8.7 mg while in candies the mg THC ranged from 0.1 mg to 39.8 mg with a median of 0.8 mg.

**Table 3:** Descriptive statistics of THC and CBD levels for each product category of cannabis edibles collected from 2014 to 2018

Product category	Number of products	Statistics	mg THC/product	mg CBD/product
Baked goods ^a^	25	Mean	24.4	6.7
		Median	8.7	0.5
		Std. deviation	29	15.7
		Range	99.1	69.1
Beverages ^b^	2	Mean	0.01	0.03
		Median	0.01	0.03
		Std. deviation	0.01	0.02
		Range	0.1	0.03
Candies ^c^	13	Mean	6.3	1
		Median	0.8	0.2
		Std. deviation	11	1.5
		Range	39.7	4.1
Chocolates	3	Mean	5	0.7
		Median	5.3	0.3
		Std. deviation	3.4	1
		Range	6.8	1.9
Frozen foods ^d*^	1	Median	–	–
		Std. deviation	–	–
		Range	–	–
Preserves ^e*^	1	Median	–	–
		Std. deviation	–	–
		Range	–	–

^a^Baked goods included brownies, bread, oatmeal cookies, chocolate chip cookies, carrot cake fruit cake, coconut choco-chip cookie, cupcakes, and Danish

^b^Beverages included coffee and grape-flavored wine

^c^Candies included gummy bear, chocolate candy, busta, kush candy, jewel candy, mango lollipop tamarind ball, lime lollipop, and peanut cake

^d^Frozen foods included ice cream

^e^Preserves included stewed June plum

*No descriptive statistics were calculated for frozen foods and preserves because of the small sample sizes

**Fig. 4:**
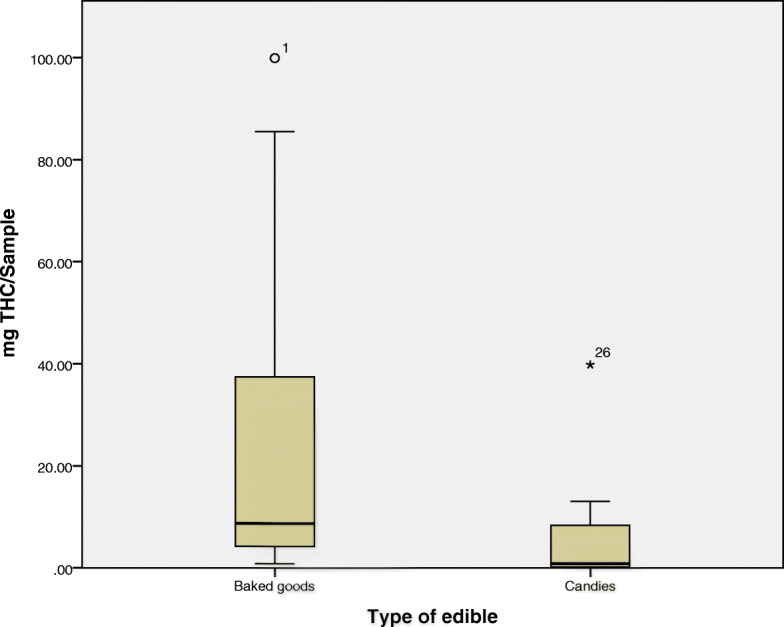
Box and Whisker plot showing the distribution of THC (mg) levels in baked goods and candies. The median is represented as line located in the middle of the box. The top and bottom of the box are the 75th and 25th percentiles respectively, and the ends of the whiskers are the 75th (or 25th) percentile ± 1.5× interquartile range. The circle represents mild outliers and the black star represents extreme outliers

For baked products, the interquartile range was 39.4, and the data were positively skewed with one mild outlier, an oatmeal cookie sample having THC levels of 99.9 mg. When the spread for candies was examined, the IQR was found to be 8.8 showing much less of a spread than the baked products and, in this category, a gummy bear candy (THC 39.8) was identified as the extreme outlier which can be seen in Fig. 4.

### Edible products from Oregon

Among the 13 candies, one was a chocolate candy which was collected from Oregon. The THC level for this sample was 8.3 mg, and the CBD level was 0.2 mg. All three chocolate bars tested were from Oregon and had higher THC to CBD ratios with THC values ranging from 1.4 to 8.3 mg/sample.

## Discussion

### Method validation

Based on the data obtained from validation, the modified QuEChERS method followed by GC-MS analysis proved useful for the extraction and quantification of cannabinoids baked products and candies. The results obtained for repeatability and accuracy satisfactorily met the internationally established acceptance criteria of ± 20% (SWGTOX 2013). Linearity was also sufficient with regression coefficients greater than 0.992.

### Edibles in Jamaica

In this study, it was found that edibles collected fell into six major categories: baked goods, candies, chocolates, frozen foods, beverages, and preserves. A study conducted in the USA revealed that brownies, cookies, and candies are among the most common food products infused with cannabis (Barrus et al. 2016). This study suggests a similar trend in Jamaica with baked goods comprising 56% and candies 29% of the total sample size respectively. The study also revealed that some local users creatively infuse cannabis into popular Jamaican sweet treats like peanut cake, busta (coconut-based hard candy), stewed June plum, and tamarind balls, which might appeal to local consumers and provide a wider base of choice for cannabis users.

### Packaging and labeling

This study revealed that none of the packages were childproof. Additionally, a common feature of all these products is that they are highly sweetened with sugar making them attractive for children who may unwittingly consume a product harmful to them. The US-based products though declaring the amount of THC on the labels were, like the local products, not presented in childproof packaging.

In the USA, packaging and labeling requirements vary across states. Cannabis is legal in Oregon and since four (4) samples were obtained from this state, their packaging and labeling requirements were reviewed.

Edible cannabis packaging and labeling requirements for Canada, where cannabis is legal, were also examined. Both Oregon and Canada had strict requirements which included: child-resistant packaging, tamper proof features, list of allergens/gluten, list of ingredients, nutritional fact panel, storage requirements, health warning messages, standardized cannabis symbol, milligrams of THC and CBD per serving, and maximum THC per serving. The major difference between the Canadian and Oregon regulations was the maximum THC per serving. For Canada, it was 10 mg per serving (Government of Canada 2019) whereas Oregon was 5 mg per serving (Oregon Liquor Control Commission 2018).

In Jamaica, packaging and labeling requirements are non-existent for cannabis edibles as it is still considered illegal. The absence of basic packaging requirements for these products brings to question the physical, chemical, and sanitary integrity of these food items. Child-proof packaging and proper labeling are imperative because these minimize the risk of unsuspecting persons, including children, consuming cannabis-infused foods which may be indistinguishable from regular and popular snacks.

### THC and CBD contents

The high THC to CBD ratio of edibles reflects a trend similar to that observed in a study being done on plant material collected in Jamaica. THC levels among all the products varied from 0.01 to 99.9 mg/sample while levels for CBD were 0.001–69.2 mg/sample.

There are several possible reasons for this variation. THC levels in the finished edible product are primarily determined by the chemical composition of the starting plant material used, and if this is not known, then the outcome of the finished product is unpredictable. The effectiveness of the method used to extract the cannabinoids will determine how much THC gets incorporated into the final product. Variation can arise since there is no standardized method of producing edibles. Finally, since cannabinoids are light and heat sensitive, improper handling and storage of the finished product could lead to degradation of the active compounds.

The observation that THC levels were higher in baked products than in candies can be explained by the fact that non-polar cannabinoids are more efficiently extracted in oil-based products like cookies than water-based ones such as candies. The sample size for preserves (n = 1) and frozen foods (n = 1) were too small for the range of THC levels to be determined.

Edibles with high CBD levels had equally high or even higher levels of THC. The production of CBD-infused edibles can be viewed favorably as CBD is a non-psychoactive cannabinoid of potentially useful therapeutic value. Like THC, the highest CBD levels were detected in cookies and brownies and the lowest in candies.

Thirteen percent of the edible products had a balanced 1:1 THC to CBD ratio. These findings are not surprising given the trend to combine THC with CBD as CBD modulates the pharmacological actions of THC thereby maximizing the therapeutic benefits while minimizing its adverse effects (Todd and Arnold 2016).

The states of Colorado and Washington, which allow recreational cannabis use, recommend a 10 mg THC per serving of edibles as of February 2015 (“Safety with edibles”, 2019), while in the state of Oregon, the allowable limit of THC is 5 mg per serving. The Colorado laws, for example, state that no product should contain greater than 100 mg THC and that products should either be subdivided in appropriate 10 mg serving sizes or instructions written on the packages as to how to achieve the recommended 10 mg dose. Three of the four chocolate products from Oregon exceeded the 5 mg limit. However, not having access to the original packaging and labeling instructions on appropriate sample size, the researchers could not determine whether these products were in breach of the Oregon regulations. It is of great concern that with the Jamaican products not being labeled and the levels of THC not declared, persons eating these high THC products are at increased risk of overdosing. This situation is further compounded if the user is unaware of the delay of onset of the psychoactive effects associated with oral ingestion. One needs to consider the potential danger of an unsuspecting person (especially children) possibly consuming 3–5 of the 99.9 mg THC cookies which translates to them having 30–50 times the recommended dosage at one sitting.

## Conclusions

There was a lack of basic packaging and labeling requirements and a wide variation in the cannabinoid content of the edibles studied in this research. This comes as no surprise since the Dangerous Drug Act Amendment in 2015 outlines no laws specifically related to edibles.

All the products mimic well-liked snacks and sweets and were not presented in child-resistant packaging, a situation that could increase the likelihood of accidental consumption by young children or entice teens into using cannabis. Levels of psychoactive THC were in few cases (13%) equal to those of CBD (non-psychoactive) with most samples (87%) having significantly higher levels of THC (range 0.1–99.9 mg per product) than CBD (0.001–69.2 mg per product). The lack of accurate labeling and the wide range of potency of edibles also raises public health concerns for adult users who are at risk of overdosing since, unlike smoking, they are unable to self-titrate the dosage.

The existing cannabis edible market in Jamaica has taken root as an unregulated, legally ambiguous system operating between the decriminalization and illegality of cannabis following the country’s 2015 cannabis reform bill. This “gray area” has resulted in the proliferation of local cannabis edible products and commerce, and, as demonstrated by this research, without effective regulation or oversight, cannabis edibles can pose a significant public health concern.

## Limitations

Prior to decriminalization in 2015, the collection of marijuana edibles was challenging, and products could hardly be sourced openly, which resulted in the collection of only two samples. It was post-decriminalization that the remaining 43 samples of the test set became readily available and the variety of products expanded. Another limitation is that convenient sampling was utilized which could have resulted in the under-representation of product categories such as frozen food, beverages, and chocolates. Convenience sampling also limits the ability to generalize the potency of cannabis edibles in Jamaica.

## Recommendations

This study provided valuable information in regard to the edibles on the Jamaican market after cannabis policy reform and has subsequently influenced policy and resulted in the Jamaican Government imposing a ban on the sale of edibles at festivals and concerts. This move was a move that was criticized by commercial producers who saw possible business opportunities emerging from the production of edibles for the tourism and the medicinal marijuana industries. Given the findings of this study, it is obvious that before any consideration on lifting the ban on edibles that a robust regulatory system be implemented that maintains the following conditions: (i) proper labeling standards are established and enforced, (ii) THC and CBD levels are declared, (iii) the manufacturing or packaging of edibles that appeal to youth are prohibited and the government regulates what is sold, (iv) childproof packaging is used in accordance with proposed standards set by the Jamaican Government, (v) potency levels are regulated, and (vi) methods for producing edibles are standardized.

The ban has no impact however on homemakers who prepare edibles for personal use or for sale in schools. Unfortunately, these cases might go undetected until they present as overdosing events at the emergency room. Though this study was focused on the potency, it would be worthwhile to conduct investigations into the possible presence of harmful contaminants such as pesticides, solvents, and mycotoxins in edibles.

There appears to be a demand for cannabis-infused edibles as an alternative to smoking in the cannabis market. If the government is to consider the decriminalization of edibles, then the sale and production by dispensaries should be regulated as cannabis buds and other cannabis-related products. Small businesses engaged in edible production should be forced to be licensed or they should channel their products through licensed strictly regulated dispensaries.

## Data Availability

The datasets used and/or analyzed during the current study are available from the corresponding author on reasonable request.

## Abbreviations

CBD: Cannabidiol
CBDA: Cannabidiolic acid
CBD-d3: Deuterated cannabidiol
CBN: Cannabinol
CBN-d3: Deuterated cannabinol
FMSTRC: Faculty of Medical Sciences Teaching and Research Complex
GC-MS: Gas chromatography-mass spectrometry
HPLC: High-pressure liquid chromatography
LOD: Limits of detection
LOQ: Limits of quantification
MSD: Mass selective detector
MSTFA: N-methyl-N-(trimethylsilyl) trifluoroacetamide
NCDA: National Council on Drug Abuse
QuEChERS: Quick, Easy, Cheap, Effective, Rugged, and Safe
THC: Delta-9-tetrahydrocannabinol
THCA: Delta 9-tetrahydrocannabinolic acid
THCA-A: Tetrahydrocannabinol acid A
THC-d3: Deuterated tetrahydrocannabinol
